# Supplementary material for: Classification of mountain-based rail transit stations and analysis of passenger flow influencing mechanisms
Source: PLoS One. 2025 May 27;20(5):e0323937. doi: 10.1371/journal.pone.0323937 (PMC12111614; doi:10.1371/journal.pone.0323937)
Supplement: Attachment — Submitted filename: Response to Reviewers.docx [file pone.0323937.s010.docx]

**To whom it may concern,**

I am writing to you regarding the modifications made to our paper (manuscript number PONE-D-24-48526: “**Classification of mountain-based rail transit stations and analysis of passenger flow influencing mechanisms** ”) in light of the reviewers’ comments.

All of the comments from the two anonymous reviewers were carefully studied and followed when we revised the paper. The details about our response to the reviewers’ comments are summarized in the attachment at the end of this letter for your convenience.

We truly appreciate the reviewers’ constructive comments and believe that the revised paper is now clearer and more useful to researchers and practitioners. If you have further questions, please feel free to contact me at **990201900066@cqjtu.edu.cn.**

Sincerely yours,

Qingru Zou

# Response to Reviewers’ Comments

Dear Reviewers,

Thanks very much for your time and valuable suggestions. The authors have carefully and seriously considered the questions and suggestions enclosed in the review and made necessary revisions.

The reviewers’ comments are in **BOLD**. The updated contents in the article are in RED.

# Reviewer 1

(1) **Figures and some of the references were missing, so it was hard to review the manuscript. Moreover, this work presents some interesting results about a specific case study but it dose not have any theoretical contribution.**

*Response:*We are sorry to bring you the confusion.As for the lack of references in the literature, we checked the full text again and found that some professional summaries were not cited. In this revision, 7 references have been added and cited, hoping to read the article better.

For the missing chart, we checked the full-text chart again, probably because Figure 4 was given directly without elaboration, leading to reading difficulties. Here we added an explanation of Figure 4.

The results of this paper can provide a basis for analyzing the passenger flow of the station in mountain cities, and provide a reference for the station to take measures to improve the passenger flow.

Modification: The new papers are added in section references. (Page22,24)

The text“Employment mountain sites are primarily distributed within the central urban areas, while employment non-mountain sites are located in the central regions of each group. Residential mountain sites are concentrated in the main urban area and its surroundings, whereas residential non-mountain sites are mainly distributed in the outskirts of each region, highlighting a pronounced separation between employment and housing. Comprehensive mountain sites are mainly situated along the South Bank and near Jiefangbei, while comprehensive non-mountain sites are predominantly located in Yuzhong and Shapingba districts. This distribution indicates that clusters closer to the urban center tend to have flatter terrain and a higher degree of land development.

Analyzing the development trajectory of administrative districts reveals potential factors contributing to the significant differences in the distribution of site types within the main urban area. The central urban group exhibits a higher degree of urbanization, concentrated development, and well-established urban functions, providing numerous employment opportunities and attracting employment-oriented sites. In contrast, development in groups outside the main city has progressed at a slower pace, with urban functions still under improvement and site development yet to fully take shape, resulting in the prevalence of residential sites in these areas. Furthermore, with regional centers being more developed than their peripheral counterparts, comprehensive stations tend to emerge in these clusters. Additionally, the unique geographical conditions and mountainous terrain of mountain cities significantly influence the spatial distribution of different station types.”is added to the paper.（Page 25）

References 12,35,37,38,39,40,41 are cited in the article.(Page 5,13,16,17,18)

# Reviewer 2

**In section 1:**

**(1) Reference sorting and citation format.**

*Response*: Thanks for your comments. Here, we reorganized the document ranking from small to large, and uniformly cited references at the end of the sentence.

*Modification:* The order of references and citations have been revised.Taking the last act of the first paragraph on page 3 as an example, we put reference 4 at the end of the sentence.(Page 3)

(2) **There is poor continuity between paragraphs, and the explanation of the three single-indicator classification approaches is vague.**

*Response*: We are sorry to bring you the confusion. Here, we have reorganized the articulation at the beginning of each paragraph and restated the three individual indicators in more detail.For better connection，we divided the literature review into two parts: site classification and regression analysis. After site classification, the regression analysis of different sites can more intuitively show the impact of different factors on different types of sites, so that corresponding measures can be made for different types of sites to improve passenger flow.

*Modification：*We have redivided site classification and regression analysis into two descriptions. At the begining of description, the “Since the characteristics of each station and its surrounding land use affect stations differently, leading to variations in passenger flow patterns, it is essential to investigate the mechanisms influencing passenger flow across different station types based on a reasonable classification of stations.” is added to the paper. The text “Wu Jiaorong (2007) categorized subway stations into distinct functional types based on their varying transfer facilities[9].” is added to the station characteristic indicators classfication. The text “In the United States, the Center for Transit-Oriented Development (2010) classified stations into 15 types by considering factors such as the number of jobs around the stations, the annual total mileage of households in the traffic area, and whether the surrounding areas were residential, employment-focused, or mixed-use [10].” is added to the land use classfication. The text “Meekyung (2018) grouped 233 subway stations in Seoul into eight categories using data on subway card swipes, passenger flow at different times, and fluctuations in passenger flow curves[11]. Similarly, Li et al. classified sites into six categories based on passenger traffic characteristics such as the number of peaks and troughs and the skewness in flow fluctuations [12].”is added to the passenger flow classfication. (Page4,5)

Two level 3 subheadings are added to the text. The subheading “1.1.1 Classification of subway stations”is added to the paper. (Page 4)

The subheading “1.1.2 Study on the influence mechanism of passenger flow”is added to the paper.(Page 5)

(3) **Pay attention to the paper alignment.**

*Response:* Thanks very much for your comment. To align the paper, this article has set the full page margins to 2cm to the left and right, and formatted the table to automatically resize according to the window.

**In section 2:**

(1) **Pay attention to the spelling of words, e.g. H0 is "the", not "he".**

*Response:* We are sorry for the improper English expression. We have checked the full text and made extensive corrections for spelling, grammar and other errors. Only the part proposed by the reviewer is shown here.

*Modification:* The word “H_0_ is he”is revised as “H_0_ is the”. (Page 8)

(2) **Pay attention to the format of the serial numbers of formulas 5, 6, and 7.**

*Response*: Thanks for the reviewer’s comment. We have checked the format requirements of other formulas, and modified the formula and serial number format including 5,6,7.

*Modification*: We adjusted the type of input method typing, and the “（）” is changed to “()”(Page 12)

(3) **Pay attention to the format of the subsequent tables on the spread.**

Modification: Thanks for the reviewer’s comment. We have modified the table style of the full text to the format required by the journal, and the table size to adjust the size by window.

(4) **Where are the eleven indicators?Not thirteen?**

*Response*: We are sorry for the mistakes. There should be 13 indicators here.

*Modification*: The "eleven" has been revised as "thirteen".(Page 9)

(5) **Pay attention to the part of speech of the table name, the "descriptive" in the second table should be changed to "description", and what exactly does the third column "abridge" refer to?**

*Response*: We are sorry for the problem. For this word error, we have made a correction in the article.The abridge stands for abbreviation

*Modification*: The "descriptive" has been revised as "description".The "abridge" has been revised as "abbreviation".（Page 11）

**In section 3**

(1) **K represents the cluster type, so how can the K value be used to describe the number of clusters?**

*Modification*: The text “K-means clustering is an iterative analysis method that achieves optimal classification by continuously adjusting the positions of cluster centers [35]. The site data is divided into K groups, randomly select K objects as the initial cluster center, and then calculate the distance between each object and each seed cluster center, and assign each object to the nearest cluster center, the cluster center and the object assigned to them represent a cluster. For each sample assigned, the cluster center of the cluster is recalculated based on the existing objects in the cluster. This process is repeated until no objects are reassigned to different clusters.”is added to the paper. (Page 13)

(2) **Put the 189 stations after visualizing Chongqing in front of your analysis to observe easily.**

*Response:* Thanks for your comments.The analysis of 189 sites in Chongqing has been added to the back of the site visualization for easy review.(Page 14)

(3) **What are the parameters used to measure VIF and Moran'I,and how does the magnitude of their values affect the results?**

*Response*: VIF is a parameter to test the correlation among influencing factors, Moran'I is a parameter to evaluate the significance of the influence, and the positive and negative values of Moran'I indicate that the influence is positively correlated or negatively correlated. The formulas for solving VIF and Moran'I parameter values are added in this paper.

*Modification*: “Only if there is no strict collinearity between the independent variables can the model analysis be conducted.The collinearity test is represented by the variance inflation factor (VIF).The greater the VIF, the higher the collinearity probability between the independent variable and other variables.VIF within 10 indicates that there is no serious collinearity[37].

The VIF of the jth independent variable is:

$\mathrm{VIF}_{j}=\frac{1}{1-{R_{j}}^{2}}$	(8)

Where:

VIF_j_ is the variance expansion coefficient of the jth independent variable; R_j_^2^ is the determination coefficient of the jth independent variable as the dependent variable, and the other independent variables are used as linear regression.

There may be spatial dependence between neighboring sites, and this influence cannot be explained by OLS regression, so it is necessary to test the spatial correlation between sites. In this study, Moran 'I was selected as the test index, and the spatial projection coordinates and influencing factors of each site were imported into Arc GIS for the spatial autocorrelation test[38].

The calculation formula of Moran'I is as follows:

$Moran'I=\frac{\sum_{i=1}^{m'} \sum_{j=1}^{m'} \gamma_{\mathrm{ij}}(e_{i}-\overline{e})(e_{j}-\overline{e})}{S^{2}\sum_{i=1}^{m'} \sum_{j=1}^{m'} \gamma_{\mathrm{ij}}}$	(9)

$\gamma_{ij}=e^{-{(\frac{d_{ij}}{b})}^{2}}$	(10)

Where:

m’ is the total number of rail transit stations;γ_ij_ is the weight between sites;e_i_ and e_j_ are represented as independent variable indicators for the i and j sites;$\overline{e}$is the mean value of site independent variable indicator e;S^2^ is the variance of the site-independent variable index e;d_ij_ is the European distance between site i and site j;

b is the bandwidth, which refers to the non-negative decay parameter of a weighted remote function.

When the sample space distribution is relatively uniform, equal bandwidth is often used, and the opposite is used.The value of Moran’I is usually [-1, 1].Under certain significance tests, the Moran index is 0.That is, there is no spatial autocorrelation. A value greater than 0 indicates that the variable has agglomeration, while a value less than 0 indicates that the variable has dispersion (spatial negative correlation) [39]. Generally, normalized statistical Z-value is used to conduct a significance test, and its expression is as follows:

$Z(Morans'I)=\frac{Morans'I-E(Morans'I)}{\sqrt{Var(Morans'I)}}$	(11)

Where:

E (Moran’I) is the theoretical mathematical expectation of the Moran’I;$\sqrt{Var(Morans'I)}$ is the theoretical variance.

The reliability of Moran’I can be evaluated by the Z-value and P-value of significance. If the significance P value is less than 0.05 (through a 95% confidence test) the absolute value of the Z score exceeds the critical value of 1.96, indicating that the results of Moran’I are credible, and more than 95% of the certainty is that the data are spatially correlated. When the value of Moran’I is greater than 0, the spatial correlation is positive; when the value of Moran’I is less than 0, the correlation is negative[40].”The text is added to the article.(Page 15,16,17)

(4) **In the description of Table 4, what does the regression results of the two models of MGWR and GWR are all greater than 3,3 come from, and what does it mean, and what does it mean that the MGWR model can be based on GWR?**

*Response*: We are sorry for the unclear description. MGWR compared with the GWR model, the difference of indexes RSS, AIC, and AICc values is greater than 3, which means that MGWR model is better.The "3" here is derived from the paper "Research on the type identification and influence mechanism of urban rail transit stations from the perspective of passenger flow characteristics". For the sake of precise description, it is rephrased that MGWR significantly outperforms GWR in all indicators, thus making the model superior.Since both GWR and MGWR can be used to describe spatial variables, AICc is an important evaluation index to evaluate whether the model is true when describing spatial variables. The smaller AICc is, the more accurate the model regression is, and the AICc value of MGWR is less than GWR, so MGWR is considered to be the model optimized and improved based on GWR model.

*Modification*: The text “Taking the AICc index as the bandwidth optimization criterion, it is found that the AICc index value of the MGWR model regression is significantly smaller than that of the GWR model in different periods, which indicates that the MGWR model realizes further optimization based on the GWR model.” is added to the paper.(Page 19)

(5) **What is the meaning of “**”in table 5?**

*Response*: We are sorry for the unclear description. The "**" symbol indicates significance in the confidence interval.

*Modification*:The text“Note: ** indicates significance on a 95% confidence interval.”is added to the bottom of the table.(Page 20)

(6) **What are the references to independent variables, global indicators,and local indicators?**

*Response*: We apologize for the lack of references. We consulted the relevant data again, and quoted the data as reference in the paper.Just as references 41,42 are cited in the paper, these documents record the meaning of independent variables, global parameters, and local parameters in the model.

*Modification*: References 41,42 have been added on page 18.(Page 18)

(7) **Please describe Figure 4 as necessary.**

*Response*: We are sorry for the unclear description. We have added the description of Figure 4，seeing reviewer 1’s response for a detailed description of Figure 4.(Page 15)

(8) **In section3.2.1 ,the last sentence is repeated, pay attention to the coherence of the sentence.**

*Response*: We are sorry for the mistakes. There is a duplicate error in the statement, which has been modified in the article, and other statements have been checked for the same error.

*Modification*: The text has been revised as“The Z-values of the 12 indicators are above 1.96, achieving the level of significance,which meets the conditions for establishing a regression model.”

**In section 4**

(1) **The content of the conclusion is cumbersome, and it briefly summarizes how to achieve this study, what conclusions have been reached, and how it can be applied to real life.**

*Response*: Thanks very much for your comment. We took this advice and summarized the conclusions of this article more succinctly and applied them to real life, listing several improvements to increase traffic to the site.

*Modification*: The text has been revised as “Based on passenger flow data and built environment data from mountain urban rail stations, this study employs the K-means clustering algorithm to classify the stations and establishes OLS, GWR, and MGWR models for regression analysis of passenger flow characteristics. The findings indicate that stations can be categorized into six types: comprehensive non-mountain, comprehensive mountain, residential non-mountain, residential mountain, employment mountain, and employment non-mountain. A comparison of model performance reveals that the MGWR model achieves better regression fitting than the GWR and OLS models. The MGWR analysis highlights that the influence of various indicators on passenger flow is spatially non-stationary and varies with geographical location. For instance, a higher density of bus lines and parking lots around a site significantly increases peak passenger flow during the morning and evening. This effect is most pronounced at employment-oriented non-mountain stations and residential non-mountain stations. To enhance passenger flow, it is recommended to increase the number of buses and parking facilities near these types of stations. Additionally, lower average road slopes and road growth coefficients are associated with higher peak passenger flow, particularly at residential non-mountain stations. Thus, improving the pedestrian environment around these stations could attract more passengers.”. (Page 20,21）

(2) **If any, the shortcomings of the study and the future direction of the study should also be mentioned.**

*Modification*: The text “This study provides a theoretical foundation for increasing the modal share of rail transit, though certain limitations remain. The passenger flow data used in this study were collected at hourly intervals. To better understand the influence of various indicators on passenger flow, future research could refine the time intervals to 15 minutes or less. Furthermore, while this study classifies POI data based on standard urban planning land use categories, subsequent research could further differentiate POI types to analyze the temporal and spatial distribution of passenger flow at urban rail transit stations with greater precision.” is added to the paper.(Page 21)
