# Supplementary material for: Classification of mountain-based rail transit stations and analysis of passenger flow influencing mechanisms
Source: PLoS One. 2025 May 27;20(5):e0323937. doi: 10.1371/journal.pone.0323937 (PMC12111614; doi:10.1371/journal.pone.0323937)
Supplement: Attachment — Submitted filename: Response_to_Reviewers_auresp_2.docx [file pone.0323937.s011.docx]

**To whom it may concern,**

I am writing to you regarding the modifications made to our paper (manuscript number PONE-D-24-48526 R1: “**Classification of mountain-based rail transit stations and analysis of passenger flow influencing mechanisms** ”) in light of the reviewers’ comments.

All of the comments from the reviewers were carefully studied and followed when we revised the paper. The details about our response to the reviewers’ comments are summarized in the attachment at the end of this letter for your convenience.

We truly appreciate the reviewers’ constructive comments and believe that the revised paper is now clearer and more useful to researchers and practitioners. If you have further questions, please feel free to contact me at **990201900066@cqjtu.edu.cn.**

Sincerely yours,

Qingru Zou

# Response to Reviewers’ Comments

Dear Reviewers,

Thanks very much for your time and valuable suggestions. The authors have carefully and seriously considered the questions and suggestions enclosed in the review and made necessary revisions.

The reviewers’ comments are in **BOLD**. The updated contents in the article are in RED.

# Reviewer

**Introduction Section**

**Literature Review:**

**(1) Page 5, section 1.1.1 — Please consider replacing the word ‘subway stations’ in your section header with ‘urban rail rapid transit stations’, as this better encapsulates above ground and below ground stations.**

*Response*: Thank you very much for your constructive feedback and suggestions on our manuscript. We have carefully reviewed your comments and have made the necessary revisions to improve the clarity and accuracy of our work.

*Modification:* In the text, the word“subway stations” has been replaced with “urban rail rapid transit stations.”(Page 4)

**Study Area:**

**(1) Page 8, section 1.2 — It is unclear to me whether the 10 referenced rail lines are all rapid transit lines, or if they include other rail modes such as commuter rail or light rail. This should be stated.**

*Response*: We apologize for not clearly elaborating on the rail transit line modes in the text. We have now added the relevant information accordingly. The supplementary content is as follows.

*Modification：*The text “ This paper focuses on the 189 stations across Chongqing's 10 operational rail transit lines: Line 1, Line 2, Line 3, Line 4, Line 5, Line 6, Line 10, Circle Line, and Guobo Line” is replaced to the This paper selects nine operational rail transit lines in Chongqing, comprising Lines 1, 4, 5, 6, 10, the Circular Line, and the Guobo Line as subway systems (high-capacity rail transit), and Lines 2 and 3, which employ the straddle-type monorail technology (medium-capacity light rail transit system). The study investigates a total of 189 stations across these lines (Page7)

**Data and methodology Section:**

**(1) Page 8, section 2.1 — As a reader, it would be helpful to understand why the authors chose the K-means clustering algorithm when there are other unsupervised classification methods available including DBSCAN, HDBSCAN, OPTICS, or Self-Organizing Maps (SOMs). Some studies have shown that these methods are more accurate classifiers than k-means, and the DBSCAN family in particular is used for spatial clustering which is often added as a component in transit studies. I do not think that the authors should have to use one of these methods, but it would be nice if they offered a deeper theoretical background justifying their choice of clustering method.**

*Response:* In considering the use of the K-means clustering algorithm for classifying mountain rail transit stations, we understand the reviewer's concerns regarding our choice of this algorithm, especially given the existence of other unsupervised classification methods such as DBSCAN, HDBSCAN, OPTICS, or Self-Organizing Maps (SOM). Below is our explanation for ultimately selecting the K-means clustering algorithm:While DBSCAN can identify clusters of arbitrary shapes, it is highly sensitive to the selection of parameters ε (neighborhood radius) and MinPts (minimum number of points). It performs poorly on high-dimensional data and is ineffective for clusters with varying densities. When the dataset exhibits significant spatial density variations, it struggles to reasonably cluster both high-density and low-density regions. The performance of HDBSCAN is heavily influenced by the parameters min_cluster_size and min_samples. Inappropriate parameter settings can lead to the identification of too few or too many clusters. It also has high computational complexity, especially on large datasets, which can be very time-consuming. The OPTICS algorithm has high computational complexity and performs poorly on large-scale datasets, with difficulties in extracting clusters. OPTICS outputs an ordered sequence of points without explicitly providing clustering results, and additional steps and methods are still required to extract true clusters from the sequence. Self-Organizing Maps (SOM) require the selection of an appropriate network structure, and improper choices can negatively impact model performance. For large-scale datasets, the training time for SOM is relatively long.Given these limitations and the specific requirements of our study, we have chosen the K-means clustering method for this research.

*Modification: We have added the following content to the original text:Current clustering methods used by scholars include K-means, DBSCAN, HDBSCAN, OPTICS, and Self-Organizing Maps (SOM). DBSCAN performs poorly on high-dimensional data and is ineffective for clusters with varying densities. When the dataset exhibits significant spatial density variations, it struggles to reasonably cluster both high-density and low-density regions. HDBSCAN is highly sensitive to parameter settings and has high computational complexity. The OPTICS algorithm also has high computational complexity, especially on large-scale datasets, and faces difficulties in extracting meaningful clusters. Self-Organizing Maps (SOM) require the selection of an appropriate network structure, and improper choices can negatively impact model performance. Although the K-means algorithm is sensitive to initial cluster centers and faces challenges in determining the optimal number of clusters (K), its simplicity, efficiency, and suitability for large-scale datasets make it a rational choice for our study. We determine the optimal number of clusters using the elbow method and improve the stability of clustering results by enhancing the initialization method. Moreover, K-means has fewer parameters, which are easy to adjust and interpret, an important advantage in practical applications. We believe that, with appropriate data preprocessing and parameter tuning, the K-means algorithm can effectively classify mountain rail transit stations and provide support for the analysis of passenger flow impact mechanisms.*(Page7)

**(2) Page 9, section 2.1.1, 1st sentence — Why were these four indicators chosen? Please justify the selection criteria used in choosing these indicators.**

*Response:* We apologize for not clearly explaining the reasons for selecting the classification indicators in the original text. This part has now been supplemented in the manuscript.

*Modification:* The text “Based on station functionality, service capacity, and environmental impacts of surrounding areas, and considering data accessibility, quantifiability, and comprehensiveness, we propose four categorized indicators as classification criteria.Development intensity indicator--reflecting urbanization levels and economic activity density in station-adjacent areas;Transfer convenience--indicating accessibility to other zones and the station’s functional role within the transportation network;Walking accessibility--measuring passenger convenience in reaching stations;Passenger flow--capturing distinct temporal characteristics of ridership during morning/evening peak periods.”is added to the paper. (Page 8)

**(3)	Page 9, section 2.1.1, Indicator 1 — What is the data source for the POI data?**

*Response*: Thank you very much for the suggestions from the reviewer. The content has been supplemented in the manuscript.

*Modification*: The text “POI data contain spatial information of the urban fabric, including attributes such as names, category classifications, and geographical coordinates (latitude and longitude)” and “The types of facilities and service functions within a 500-meter radius around the stations significantly influence the attractiveness and vitality of the transit stations[26]. This study employs Gaode Map to obtain POI data within a 500-meter radius around rail transit stations in nine districts of Chongqing, namely Yuzhong, Shapingba, Jiangbei, Yubei, Nan'an, Jiulongpo, Dadukou, Banan, and Beibei ”are added to the paper.(Page 9)

**(4) Page 9, section 2.1.1, Indicator 3 — Why was 500 meters chosen as the threshold value for the accessibility analysis? Is this based off of travel survey data or other measurements of passenger willingness to walk to a station?.**

Modification: Thanks for the reviewer’s comment. This study reviewed relevant literature and conducted research within a 500-meter radius of rail transit stations. The characteristics of walking within 500 meters have been elaborated and supplemented in the text.

*Modification*: The text “ The 500-meter radius around rail transit stations is generally regarded as the core area for pedestrian accessibility. Within this range, residents and passengers can conveniently reach the stations on foot without relying on other transportation modes. The characteristics of roads and buildings within this range directly influence the walking experience and travel efficiency of passengers, making it a focal area for research.”is added to the paper.(Page 9)

**(5) Page 12, section 2.2.1, Indicator 1 — If this indicator is meant for “properties of the station itself”, then why would the indicator include “whether [a station] is close to a mega commercial area” as a factor? This is a built environment variable. Also, what constitutes a “large transfer station”? Is this based off of passenger flow statistics?**

*Response*: We apologize for the inaccurate naming of the indicators. The term “properties of the station itself” has been revised to “Station functional attributes”, which describes the functional characteristics of the stations, such as whether the station is near a large commercial area, whether it is a major transfer station, and whether it is an external transportation hub. These are distinguished from the built environment surrounding the station. Yes, major transfer stations are identified based on passenger flow statistics.

*Modification*: The term“properties of the station itself” has been revised to “Station functional attributes”.The original text lacked relevant descriptions, which have now been revised to:“Based on passenger flow statistics, this study designates stations with particularly high passenger volumes at transfer points as major transfer stations. The geographical location of the station is used to determine whether it serves as an external transportation hub and whether it is in proximity to a large commercial area. These three indicators are collectively referred to as the functional attribute indicators of the station.”(Page 12)

**(6) Page 13, section 2.2.2 — As a reader I would personally find it more helpful for the authors to state the independent and dependent regression variables being used in the modeling. It is not all that helpful to show a generic OLS formula. I would like to see how this formula is being applied by the authors to advance the research question. I provide the same feedback for the GWR model, all variables should be discussed in the context of the additional spatial component of the model.**

*Response*: Thank you very much for your valuable comments.We have added descriptions of the independent and dependent variables in Section 2.2.2 (now Section 2.2.3). The application of OLS and GWR in this study is reflected in the model fit comparison with the MGWR model, which is detailed in Section 3.2.2. In Section 3.2.2, after fitting the independent variables of different influencing indicators with passenger flow as the dependent variable, we found that the MGWR model has a better fit. To better analyze the influencing factors, we mainly discuss the specific impacts of different independent variables on the dependent variable through MGWR modeling in the subsequent text. The relationship between the independent and dependent variables in the MGWR model is elaborated in Section 3.2.3.

*Modification*: The text “In the regression models, the dependent variables include the total daily passenger flow, morning peak inbound passenger flow, morning peak outbound passenger flow, evening peak-hour inbound passenger flow, and evening peak outbound passenger flow. The independent variables are the station functional attributes and built environment indicators introduced in the previous section.”is added ito the text.（Page 16）

**(7) Page 13, section 2.2.2 — Passenger flow data, to my understanding, is a form of discrete count data demonstrating the number of passengers boarding or alighting at a station. Such data should generally be modeled using count models, such as those in the Poisson family (Poisson, Negative Binomial) or their spatial counterparts. Can the authors please justify their selection of OLS and its spatial counterparts (GWR and MGWR)? The authors only cite one example of OLS being used by Loo et al. in the literature review. The properties of the flow data is also not deeply explained in section 2.2.1. Perhaps it is not a count type data, which would then justify the use of OLS. I would appreciate clarity from the authors on this subject.**

*Response*: We fully understand the reviewer's concerns and apologize for not clarifying this in the original text. We have now reorganized and explained it thoroughly.While passenger flow data is generally a discrete count, in practical applications—such as in this study where the passenger flow values are large and the research objective is to analyze the impact of different factors on passenger flow rather than precisely counting the number of passengers—it can be treated as a continuous variable. Many scholars have used regression models to analyze the factors influencing passenger flow at rail transit stations. Models from the Poisson family (Poisson, Negative Binomial) or their spatial counterparts are typically used when the objective is to estimate passenger arrival probabilities.The research content of this chapter is to analyze the impact mechanisms of passenger flow at different types of rail transit stations. We use various influencing factors as independent variables in regression models to analyze their effects on passenger flow. Therefore, passenger flow data is used as the dependent variable in the regression models. Given this context, it is reasonable to use passenger flow data as the dependent variable in the OLS, GWR, and MGWR regression models in this study.

*Modification*: We have also added the following examples of researchers in the literature review section. The text “An et al. used OLS regression analysis and found that commercial land use, bus stations, and tourist attractions have significant positive impacts on Shanghai’s rail transit passenger flow, independent of weekdays.”is added to the paper. Additionally, in Section 2.2.3, we have added explanations for passenger flow data. The text “Ordinary Least Squares (OLS), Geographically Weighted Regression (GWR), and Multiscale Geographically Weighted Regression (MGWR) models are commonly used to handle continuous dependent variables. When analyzing the factors influencing station passenger flow in regression models, passenger flow data can be treated as a continuous variable. In the regression models, the dependent variables include total daily passenger flow, morning peak-hour inbound passenger flow, morning peak-hour outbound passenger flow, evening peak-hour inbound passenger flow, and evening peak-hour outbound passenger flow. The independent variables are the station functional attributes and built environment indicators introduced in the previous section.” is added to the paper. (Page 13)

**Analysis of Results Section**

**(1) Page 15, section 3.1, cluster 1 — What are ‘composite-type’ stations? This is a very unusual terminology. From my understanding, these stations have the highest passenger volumes, but I am not sure what the word ‘composite’ has to do with passenger volumes.**

*Response:* We apologize for the confusion in terminology. The term “composite-type stations” referred to “Comprehensive-type stations,” which has now been corrected in the text. Comprehensive-type stations are mixed-use stations characterized by a high proportion of both employment and residential land use, as well as intensive development. These stations attract a large volume of passenger flow due to their diverse functional attributes.(Page 14)

*Modification*: The text “composite-type stations” referred to “Comprehensive-type stations”.

**(2) Pages 17-20, section 3.2.1 — The authors should have explained their use of VIF and Moran’s I as diagnostics in the methods section, it is unusual that these formulas are being introduced and discussed methodologically in the results section. Also, the use of European Distance Band for the Moran’s I needs to be justified as this can have major implications on the precision of the findings.**

*Response*: Thank you very much for your valuable comments. We have revised the original text by transferring the discussion of VIF and Moran’s I from the conclusion section to Section 2.2.2, where the methodology is discussed.The Moran’s I index is a global measure of spatial autocorrelation, reflecting the degree of similarity between a given indicator and its spatially adjacent units. Before calculating Moran’s I, it is necessary to introduce the concept of a spatial weights matrix, which reflects the spatial proximity between multiple locations. In this study, Euclidean distance is used as the basis for assigning weights in the spatial weights matrix for Moran’s I. The product of the weight matrix γ and the independent variable indicators in the Moran’s I formula reflects the similarity between spatial units.Euclidean distance, which measures the straight-line distance between two points, accurately represents the actual distance between them in geographical space. Using Euclidean distance as the basis for weight assignment ensures that the calculation of Moran’s I accurately reflects spatial autocorrelation. Therefore, it is reasonable to use Euclidean distance as the basis for weight assignment in Moran’s I. It allows for an accurate calculation of the similarity between stations, thus providing a reliable basis for classifying mountain rail transit stations and analyzing the mechanisms influencing passenger flow.

.

Modification: The text “Prior to the calculation of the Moran’s I index, the concept of a spatial weights matrix must be introduced. This matrix reflects the spatial proximity between multiple locations by representing the relationships among different spatial units. In the context of the Moran’s I index, the spatial weights matrix is often constructed using Euclidean distance as the basis for assigning weights. The product of the weight matrix γ and the explanatory variable indicators, as shown in Equation (10), reflects the degree of similarity between spatial units. Euclidean distance, which measures the straight-line distance between two points, provides an accurate representation of the actual distance between them in geographical space and thus serves as a valid basis for assigning weights.” is added to the article.(Page 14)

`
